# Age-associated radiomic phenotyping in mice using multi-energy photon-counting and preclinical CT: an exploratory study

**DOI:** 10.1007/s10522-026-10524-y

**Published:** 2026-10-15

**Authors:** Noah A. Castioni, Carmen Wängler, Carolin Mogler, Björn Wängler, Caroline Cassia, Henning Rudolf, Matthias F. Froelich, Stefan O. Schoenberg, Alexander Hertel

**Affiliations:** 1 Department of Radiology and Nuclear Medicine, University Medical Centre Mannheim, Theodor-Kutzer-Ufer 1-3, 68167 Mannheim, Germany; 2 Biomedical Chemistry, Department of Clinical Radiology and Nuclear Medicine, Medical Faculty, Mannheim of Heidelberg University, 68167 Mannheim, Germany; 3 Institut Für Pathologie Und Pathologische Anatomie, School of Medicine and Health, Technische Universität München, Trogerstraße 18, 81675 Munich, Deutschland; 4 Molecular Imaging and Radiochemistry, Department of Clinical Radiology and Nuclear Medicine, Medical Faculty, Mannheim of Heidelberg University, 68167 Mannheim, Germany; 5 Research Campus M2OLIE, Medical Faculty Mannheim of Heidelberg University, 68167 Mannheim, Germany; 6 Department of Diagnostic and Interventional Radiology and Nuclear Medicine, Center for Radiology and Endoscopy, University Medical Center Hamburg-Eppendorf (UKE), Hamburg, Germany

**Keywords:** Photon-counting CT, Radiomics, Age-group classification, Aging, Spectral imaging, Quantitative imaging biomarkers

## Abstract

To investigate whether CT-derived radiomic features capture imaging patterns associated with chronological age groups in mice and to assess the influence of imaging platform and photon-counting CT reconstruction strategy on classification performance. Female C57BL/6NRj mice from three chronological age groups (28, 88, and 99 weeks; n = 11) underwent clinical photon-counting CT (PCCT) and preclinical Albira II CT. Heart, liver, and spleen were segmented and radiomic features were extracted. The individual mouse was defined as the independent biological unit. Model development and evaluation used nested leave-one-mouse-out cross-validation with all preprocessing, feature selection, model selection, and, where applicable, reconstruction-energy selection restricted to the respective training folds. Organ-level class probabilities were averaged to generate one mouse-level prediction. Monoenergetic PCCT reconstructions from 40 to 180 keV were evaluated, including a fully nested energy-and-pipeline selection analysis. A grouped mouse-level permutation analysis with 200 permutations was performed for the primary monoenergetic workflow. Albira II correctly classified 5/11 mice (45.5%; 95% CI, 21.3–72.0%), whereas PCCT-SPP correctly classified 10/11 mice (90.9%; 95% CI, 62.3–98.4%). The fully nested monoenergetic PCCT workflow correctly classified 9/11 mice (81.8%; 95% CI, 52.3–94.9%; balanced accuracy, 83.3%; macro-F1, 83.3%). Energy-specific performance varied considerably: 110 keV showed the highest accuracy (81.8%) and was selected in 7/11 outer folds, whereas the highest macro one-vs-rest AUC occurred at 130 keV (0.867). None of the 200 permuted analyses reached the observed fully nested monoenergetic accuracy (plus-one corrected Monte-Carlo p = 0.005). CT-derived radiomic features showed chronological age-group-associated patterns in this exploratory murine cohort, with classification performance varying substantially across imaging platforms and reconstruction strategies. PCCT-SPP showed the numerically highest performance, while monoenergetic PCCT demonstrated pronounced energy dependence without evidence for a universally optimal reconstruction energy. These findings require validation in larger independent cohorts and correlation with independent biological-aging reference measures.

**Supplementary Information:**

The online version contains supplementary material available at https://doi.org/10.1007/s10522-026-10524-y.

## Introduction

Aging is a progressive and multifaceted biological process characterized by the gradual accumulation of molecular and cellular damage over time, leading to a decline in physiological integrity, diminished regenerative capacity, and increased susceptibility to disease and death (Guo et al. 2022). This complex interplay of genetic, epigenetic, metabolic, and environmental factors drives the aging phenotype and manifests across virtually all organ systems. With global life expectancy rising, aging populations are becoming a major challenge for healthcare systems worldwide. Traditional approaches to quantifying aging have relied primarily on chronological age, which often fails to capture the heterogeneity of biological aging among individuals. Age is therefore not only a chronological parameter but also a key risk factor for a multitude of diseases, including cardiovascular disease, cancer, neurodegeneration, and metabolic disorders. Importantly, the biological age of individuals can differ substantially from their chronological age, underlining the need for accurate, individualized markers of aging (Fekete et al. 2024).

In this context, there is growing interest in developing reliable and non-invasive biomarkers of aging that can aid in early disease detection, risk stratification, longitudinal monitoring, and personalized preventive strategies. Among these, imaging biomarkers offer unique advantages by providing structural and functional information across multiple organ systems in vivo (Alikhani et al. 2024). Computed tomography (CT), in particular, enables the assessment of tissue morphology and density at high spatial resolution and has been increasingly explored for its potential to capture age-related changes in organs and tissues (Chambers 2023; Hertel et al. 2025).

Recent advancements in photon-counting computed tomography (PCCT) technology have substantially expanded the diagnostic potential of both clinical and preclinical imaging. In contrast to conventional energy-integrating detectors, PCCT systems provide superior spatial resolution, reduced electronic noise, and energy-resolved imaging capabilities, facilitating the reconstruction of monoenergetic images. These reconstructions enhance tissue contrast and enable material differentiation, thus offering considerable promise for advanced quantitative imaging applications (Willemink et al. 2018; van der Bie et al. 2025).

Concurrently, radiomics, defined as the high-throughput extraction of quantitative imaging features, has emerged as a powerful methodology for identifying subtle and complex imaging patterns that may reflect underlying pathophysiological processes. In this study, radiomic signatures derived from two distinct CT imaging platforms—a clinical photon-counting CT system and a dedicated preclinical CT system—were compared to investigate platform- and reconstruction-dependent differences in age-associated radiomic phenotypes. Although identical preprocessing, including standardized in-plane spatial resampling, was applied before radiomic feature extraction, the two imaging systems differ in multiple technical characteristics beyond detector architecture. Accordingly, the comparison should be interpreted as a platform- and protocol-level comparison rather than as an isolated assessment of photon-counting versus energy-integrating detector technology. However, the present study does not aim to estimate biological age itself. Rather, it investigates whether radiomic signatures differ across defined chronological age groups in a controlled murine aging model.

## Materials and methods

### Imaging

Three age groups of C57BL/6NRj female mice, young (28 weeks, three mice), middle-aged (88 weeks, four mice), and aged (99 weeks, four mice), were scanned groupwise using two CT systems: a clinical Photon-Counting CT (Siemens Healthineers NAEOTOM Alpha®) and a preclinical Animal CT (Albira II). The C57BL/6NRj substrain represents an inbred C57BL/6N line with a well-characterized and relatively uniform genetic background, making it a widely used reference model for aging and metabolic studies. Across the adult life span of C57BL/6N mice, age-related changes in body composition, metabolism, and organ integrity have been described, providing a biological basis for using distinct age groups to capture different stages of the aging process in this model (Petr et al. 2021). The standard PCCT spectral post-processing (SPP) reconstruction was generated with a 0.6-mm slice thickness and Qr44 kernel. In addition, virtual monoenergetic reconstructions from 40 to 180 keV were generated using the Siemens Syngo.Via MM Oncology tool. The Albira II scans used 120 kV and 400 μA at 50 μm in-plane resolution.

### Segmentation and extraction of radiomics features and statistical analysis

**Fig. 1:**
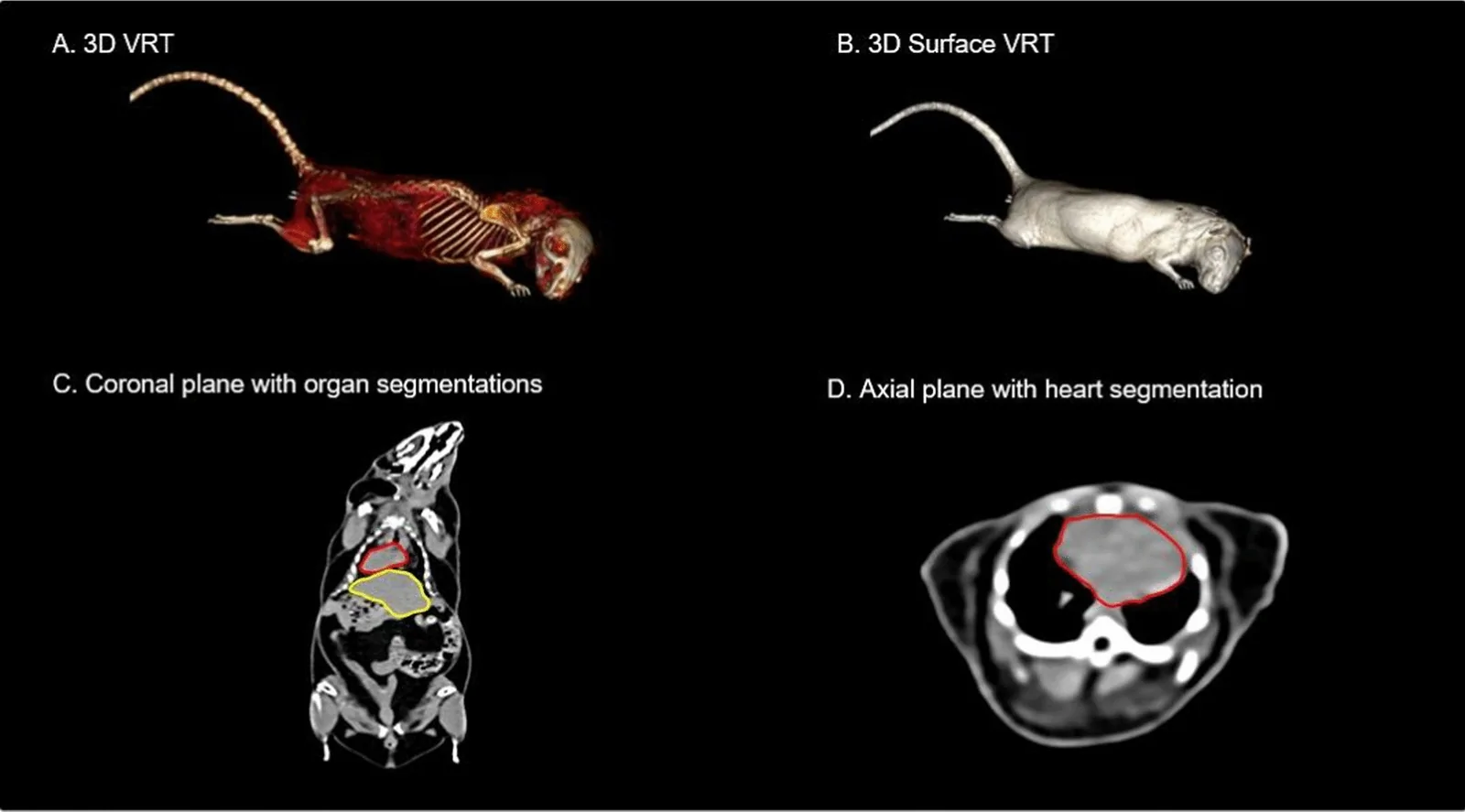
Visualization of Heart, liver, and spleen segmentation procedures in mouse CT Imaging

Manual segmentation of the heart, liver, and spleen was performed for all mice in MITK Workbench (v2024.12) by one radiologist with two years of segmentation experience. All segmentations were subsequently independently reviewed by a second radiologist for anatomical plausibility and segmentation quality. Where necessary, segmentations were corrected before radiomic feature extraction. Segmentations were done using the axial reconstructions with a soft tissue kernel and 3D masks were exported as NIfTI files. Representative segmentations of the heart, liver, and spleen are shown in Fig. 1.

Radiomic feature extraction was performed using PyRadiomics v3.0.1 (van Griethuysen et al. 2017). Images and segmentation masks were processed in three dimensions (force2D = False). Prior to feature extraction, in-plane resampling to 2 × 2 mm was performed using SimpleITK linear interpolation, while the original through-plane spacing was retained (resampledPixelSpacing = [2, 2, 0]). Consequently, the resulting voxel spacing was 2 × 2 × 0.125 mm^3^ for Albira II and 2 × 2 × 0.6 mm^3^ for PCCT. No intensity normalization was applied, and CT attenuation values were analyzed on the Hounsfield-unit scale. Gray-level discretization was performed using a fixed bin width of 25 HU, and the voxel-neighborhood distance for texture-matrix calculation was set to 1.

Radiomic features were extracted from the original image as well as from Laplacian-of-Gaussian (LoG)-filtered images with σ = 3.0 and 5.0 mm and from all eight three-dimensional wavelet decompositions. Enabled feature classes comprised shape, first-order, gray-level co-occurrence matrix (GLCM), gray-level run-length matrix (GLRLM), gray-level size-zone matrix (GLSZM), gray-level dependence matrix (GLDM), and neighboring gray-tone difference matrix (NGTDM) features. Shape features were calculated from the original image only. No explicit minimum ROI voxel count was specified (minimumROISize = None), minimumROIDimensions was set to 1, and no custom geometry tolerance was defined. The complete extraction yielded 1,015 radiomic predictor features per image/ROI: 105 features from the original image, 182 features from the two LoG-filtered images, and 728 features from the eight wavelet decompositions. PyRadiomics diagnostic outputs and metadata were excluded from the predictor set.

Initial exploratory assessment of radiomic feature variation according to imaging platform, age group, and organ was performed using principal component analysis (PCA).

### Nested cross-validation and training-fold-specific preprocessing

**Fig. 2:**
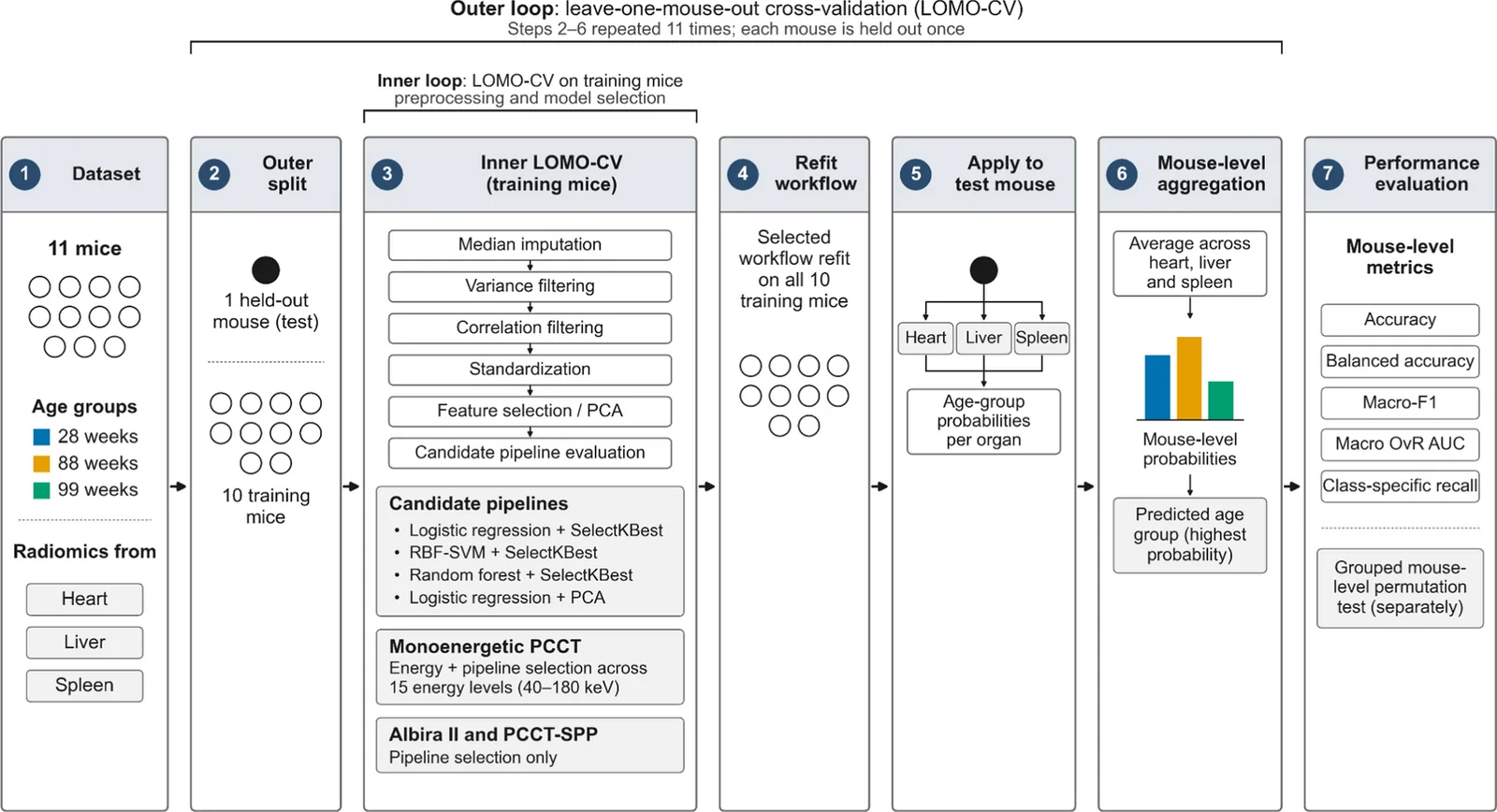
Nested leave-one-mouse-out cross-validation workflow for mouse-level age-group classification. The individual mouse was defined as the independent biological unit. In each of 11 outer LOMO-CV folds, all observations from one mouse were withheld as the test set, while the remaining 10 mice were used for model development. Within the outer-training set, preprocessing steps, including median imputation, variance filtering, correlation filtering, standardization, and feature selection or PCA, were refitted within an inner mouse-grouped LOMO-CV procedure. Candidate analytical pipelines were compared using the inner training folds. For Albira II and PCCT-SPP, pipeline and hyperparameter selection were performed within the inner loop; for the primary monoenergetic PCCT analysis, reconstruction energy and analytical pipeline were jointly selected from the training data. The selected workflow was subsequently refitted using all 10 outer-training mice and applied once to the held-out mouse. Organ-level class probabilities from heart, liver, and spleen were averaged to obtain one mouse-level probability vector and one final age-group prediction per outer fold. Performance metrics were calculated from the 11 resulting out-of-fold mouse-level predictions. Grouped mouse-level permutation testing was performed separately by repeating the complete analytical workflow under permuted age-group labels

The individual mouse was defined as the independent biological unit throughout model development and evaluation. Heart, liver, and spleen measurements were treated as repeated observations nested within each mouse. Model evaluation was performed using nested leave-one-mouse-out cross-validation (LOMO-CV; Bradshaw et al. 2023). The outer loop comprised 11 folds, with all observations belonging to one mouse withheld as the independent outer test set in each fold. Model and preprocessing decisions were based exclusively on the remaining 10 mice. Within each outer-training set, an inner mouse-grouped LOMO-CV procedure was used for model and pipeline selection. The nested analytical workflow is summarized schematically in Fig. 2.

All data-dependent preprocessing steps were fitted exclusively on the respective training partition. Missing numerical values were imputed using medians estimated from the training data only. Features with essentially zero variance were removed using a variance threshold of 1 × 10⁻^12^. Pairwise feature correlations were subsequently calculated within the training data, and redundant features with an absolute correlation coefficient >0.90 were removed. Retained features were standardized using the mean and standard deviation derived from the corresponding training partition.

For supervised feature selection, SelectKBest using ANOVA F-statistics (f_classif) was fitted exclusively to the training labels, and the selected features were subsequently applied unchanged to the corresponding validation or test data. When principal component analysis (PCA) was used, PCA was fitted only to standardized training data, and validation or test observations were projected using the transformation learned from that training partition. During inner cross-validation, all preprocessing steps were independently refitted within each inner-training fold.

Candidate model configurations were compared exclusively within the inner cross-validation procedure. After selection of the best-performing pipeline, the complete preprocessing and modeling workflow was refitted on all outer-training mice and applied once to the held-out mouse. For monoenergetic PCCT analyses, reconstruction-energy selection, where applicable, was likewise performed exclusively within the outer-training data. At no stage were observations from the held-out outer test mouse used to estimate imputation values, variances, correlations, standardization parameters, SelectKBest scores, PCA components, model hyperparameters, classifier choice, or reconstruction-energy selection.

### Unit of analysis and mouse-level prediction

The individual mouse was considered the independent biological unit (n = 11). Heart, liver, and spleen measurements and, where applicable, multiple monoenergetic reconstructions were treated as repeated measurements nested within each mouse and were not considered independent samples. For each held-out mouse, organ-level class probabilities were averaged across heart, liver, and spleen to obtain one mouse-level probability vector. The class with the highest averaged probability was assigned as the final mouse-level prediction. Thus, each outer LOMO-CV fold contributed exactly one independent prediction.

### Model selection and hyperparameter optimization

For the Albira II and PCCT-SPP analyses, model selection and hyperparameter optimization were performed within the inner cross-validation loop of each outer LOMO-CV fold. To limit model-selection complexity given the small number of independent animals, a compact prespecified search space was used. Candidate pipelines comprised Logistic Regression with SelectKBest, RBF-SVM with SelectKBest, Random Forest with SelectKBest, and Logistic Regression with PCA.

For Logistic Regression with SelectKBest, the number of selected features was varied between k = 3 and k = 5, and the regularization parameter C between 0.1 and 1.0. Class weights were balanced, the maximum number of iterations was set to 5,000, and random_state was fixed at 42. For RBF-SVM with SelectKBest, k = 3 or 5 and C = 0.1 or 1.0 were evaluated; gamma was set to “scale”, class weights were balanced, probability estimates were enabled, and random_state was set to 42. For Random Forest with SelectKBest, k = 3 or 5, maximum tree depth = 3 or 8, and minimum samples per leaf = 1 or 2 were evaluated; the number of trees was fixed at 50, minimum samples required for splitting at 2, class_weight at “balanced_subsample”, and random_state at 42. For Logistic Regression with PCA, PCA retained either 80% or 90% of the explained variance and C was varied between 0.1 and 1.0; class weights were balanced and the maximum number of iterations was set to 5,000. The complete candidate search space and fixed model settings are summarized in Supplementary Table S3.

Within each outer fold, all candidate configurations were evaluated using inner mouse-grouped LOMO-CV. Candidate configurations were ranked according to inner mouse-level classification accuracy. In the event of identical performance, ties were resolved deterministically according to the prespecified grid order. The selected pipeline and parameter configuration were subsequently refitted using all outer-training mice before being applied to the held-out mouse.

### Albira II sensitivity analysis

A secondary sensitivity analysis was performed for the Albira II dataset to assess the influence of higher-dimensional feature-selection and dimensionality-reduction strategies. This analysis was separate from the primary adaptive nested model-selection workflow. A fixed XGBoost classifier (XGBClassifier) was used with 50 estimators, a maximum tree depth of 2, a learning rate of 0.10, subsample = 0.80, colsample_bytree = 0.80, L2 regularization (reg_lambda) = 1.0, objective = “multi:softprob”, num_class = 3, eval_metric = “mlogloss”, random_state = 42, n_jobs = 1, and verbosity = 0. No early stopping or explicit class weighting was applied; parameters not specified above remained at the respective library defaults.

Each prespecified configuration was evaluated separately using outer LOMO-CV without additional hyperparameter optimization. Within each training fold, median imputation, near-zero-variance filtering (variance ≤ 1 × 10⁻^12^), correlation filtering (|r|> 0.90), and standardization were refitted using the training data only. The compared configurations comprised correlation-filtered radiomics without additional dimensionality reduction, PCA retaining 5, 10, 15, or 20 components, and SelectKBest using ANOVA F-statistics (f_classif) retaining 10, 20, 30, or 50 features. PCA and SelectKBest were likewise fitted exclusively within the respective training fold. The number of features remaining after correlation filtering varied across folds, with a median of 327 features.

In each outer fold, the model was trained using the 10 training mice (30 organ-level observations) and evaluated on the three organ measurements of the held-out mouse. Organ-level class probabilities were averaged to obtain one mouse-level probability vector, and performance was calculated from the resulting 11 independent mouse-level predictions. Results of this secondary sensitivity analysis are provided in Supplementary Table S1.

### Monoenergetic PCCT model and energy selection

For the monoenergetic PCCT analysis, a deliberately compact set of four prespecified candidate pipelines was used to reduce the risk of excessive model tuning: (1) Logistic Regression with SelectKBest (k = 5, C = 1.0), (2) RBF-SVM with SelectKBest (k = 5, C = 1.0, gamma = “scale”), (3) Random Forest with SelectKBest (k = 5, 100 trees, max_depth = 3, min_samples_split = 2, min_samples_leaf = 1), and (4) Logistic Regression with PCA retaining 90% of explained variance (C = 1.0). Hyperparameters were fixed for these spectral candidate pipelines; only pipeline selection was performed within the inner cross-validation loop.

Each monoenergetic reconstruction level from 40 to 180 keV was first evaluated separately using nested LOMO-CV. In addition, a fully nested spectral analysis was performed in which both reconstruction energy and analytical pipeline were selected exclusively within the outer-training data. For each outer fold, the 15 reconstruction energies were crossed with the four candidate pipelines. Inner mouse-grouped LOMO-CV among the 10 training mice was used to identify the energy/pipeline combination with the highest inner mouse-level accuracy. The selected combination was then refitted on all outer-training mice and applied once to the held-out mouse. This fully nested energy-and-pipeline selection constituted the primary monoenergetic PCCT analysis.

### Feature-selection stability analysis

For the 110-keV reconstruction, feature-selection stability was assessed across the 11 outer LOMO training folds. Within each fold, correlation filtering and SelectKBest (k = 5) were fitted exclusively using the corresponding training mice. The frequency with which each feature was selected across outer folds was recorded as a descriptive measure of selection stability.

### Performance assessment

Classification performance was assessed exclusively at the mouse level using the out-of-fold predictions generated by the 11 outer LOMO-CV folds. Primary performance metrics included overall accuracy, balanced accuracy, macro-averaged F1 score, and class-specific recall. For overall accuracy, 95% confidence intervals were calculated using the Wilson score method.

Receiver operating characteristic (ROC) analysis was performed using the out-of-fold mouse-level class probabilities. One-vs-rest ROC curves and corresponding AUC values were calculated separately for each age group, and macro one-vs-rest AUC was used as the summary measure of multiclass discrimination. Given the limited number of independent mice (n = 11), AUC estimates were interpreted descriptively and not as evidence of externally validated discrimination.

### Mouse-level permutation analysis

To assess whether the observed classification performance could arise from model optimization under random class assignment, a grouped permutation analysis was performed for the primary fully nested monoenergetic PCCT workflow. Age-group labels were permuted at the mouse level while preserving the original group sizes of three, four, and four mice. For each of 200 permutations, the complete nested analytical workflow was repeated, including training-fold preprocessing, feature selection, inner pipeline selection, and reconstruction-energy selection. The empirical null distribution of mouse-level accuracy was compared with the observed accuracy. The Monte-Carlo p-value was calculated using the plus-one correction as (b + 1)/(B + 1), where b denotes the number of permutations with accuracy greater than or equal to the observed value and B = 200 denotes the total number of permutations.

## Results

### Radiomics feature exploration and dimensionality reduction

**Fig. 3:**
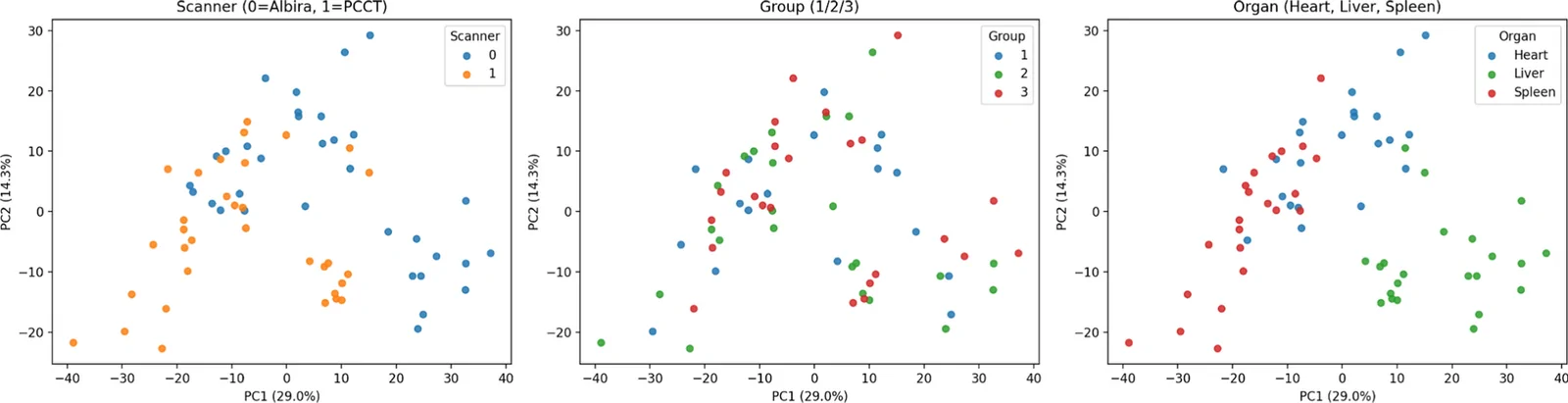
Principal component analysis visualization of radiomic feature variation by scanner, experimental group, and organ

Principal component analysis (PCA) of the unfiltered radiomics feature set extracted from segmented heart, liver, and spleen revealed clear clustering patterns related to scanner type, organ identity, and mouse age group. Scanner-dependent differences produced distinct yet partially overlapping clusters between the photon-counting CT (PCCT) and micro-CT (Albira II) data (Fig. 3). Age-group-associated patterns were also observed across individual organs, motivating subsequent exploratory predictive modeling.

### Feature selection and model performance

**Fig. 4:**
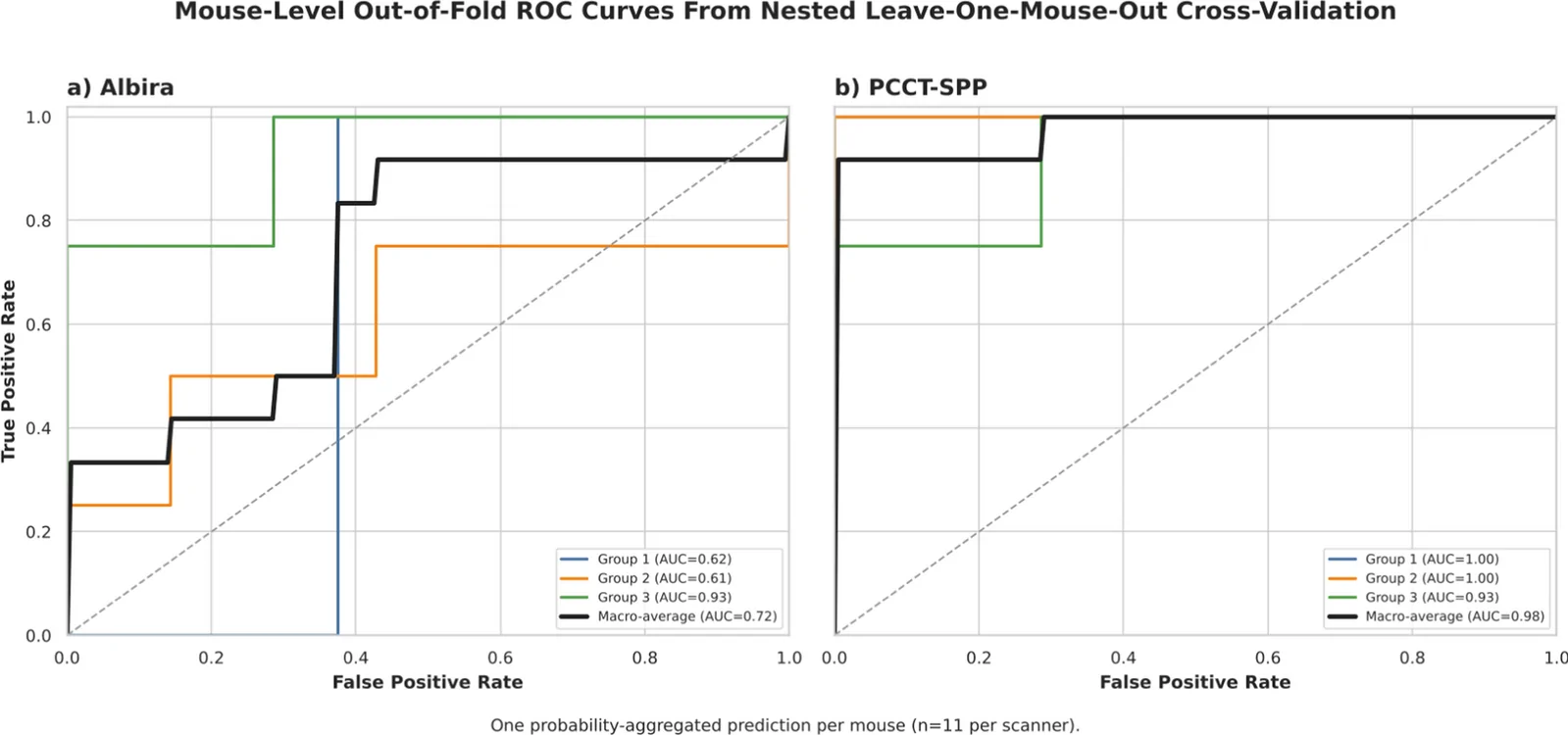
Mouse-level out-of-fold receiver operating characteristic (ROC) curves for age-group classification using Albira II and PCCT spectral post-processing (SPP) radiomics. Curves were generated from nested leave-one-mouse-out cross-validation. In each outer fold, all organ-level measurements from one mouse were withheld, while preprocessing, feature selection or dimensionality reduction, model selection, and hyperparameter optimization were performed exclusively using the remaining mice. Organ-level class probabilities from heart, liver, and spleen were averaged to obtain one mouse-level probability vector. Separate one-vs-rest ROC curves are shown for Groups 1–3 together with the macro-average ROC curve. PCCT-SPP showed numerically higher discrimination than Albira II in this cohort

Classification performance was evaluated at the mouse level using the 11 out-of-fold predictions generated by nested LOMO-CV. For the Albira II dataset, the nested workflow correctly classified 5 of 11 mice, corresponding to an accuracy of 45.5%, a balanced accuracy of 41.7%, and a macro-F1 score of 37.2%. In contrast, the corresponding PCCT-SPP workflow correctly classified 10 of 11 mice, yielding an accuracy of 90.9%, a balanced accuracy of 91.7%, and a macro-F1 score of 91.5%. The only PCCT-SPP misclassification involved one Group 3 mouse classified as Group 2. Detailed mouse-level performance metrics, including 95% confidence intervals and class-specific recall, are summarized in Table 1. Corresponding mouse-level out-of-fold ROC curves for Albira II and PCCT-SPP are shown in Fig. 4.

**Table 1:** Primary mouse-level age-group classification performance under nested leave-one-mouse-out cross-validation

Workflow	Correct mice/11	Accuracy (95% CI)	Balanced Accuracy	Macro-F1	Group 1 recall	Group 2 recall	Group 3 recall
Albira II	5/11	45.5% (21.3–72.0%)	41.7%	37.2%	0%	50%	75%
PCCT-SPP	10/11	90.9% (62.3–98.4%)	91.7%	91.5%	100%	100%	75%
Fully nested monoenergetic PCCT	9/11	81.8% (52.3–94.9%)	83.3%	83.3%	100%	75%	75%

Performance metrics were calculated from the 11 out-of-fold mouse-level predictions obtained using nested LOMO-CV. Accuracy confidence intervals represent 95% Wilson score intervals. Balanced accuracy, macro-F1, and class-specific recall were calculated at the mouse level. Group 1, Group 2, and Group 3 correspond to mice aged 28, 88, and 99 weeks, respectively. Primary SelectKBest-based candidate pipelines were deliberately restricted to k = 3 or k = 5 features to limit model complexity relative to the small number of independent animals

Inner model selection differed across outer folds. For PCCT-SPP, Logistic Regression with SelectKBest was selected in six outer folds and Logistic Regression with PCA in five folds, whereas neither SVM nor Random Forest was selected as the optimal inner configuration. For Albira II, several different pipelines were selected across outer folds, reflecting greater instability of model selection in this dataset. These results indicate substantial platform- and protocol-dependent differences in classification performance but should not be interpreted as evidence for an isolated effect of detector technology. The higher-dimensional Albira feature-selection and dimensionality-reduction sensitivity analysis is provided in Supplementary Table S1.

### photon-counting CT spectral analysis

**Fig. 5:**
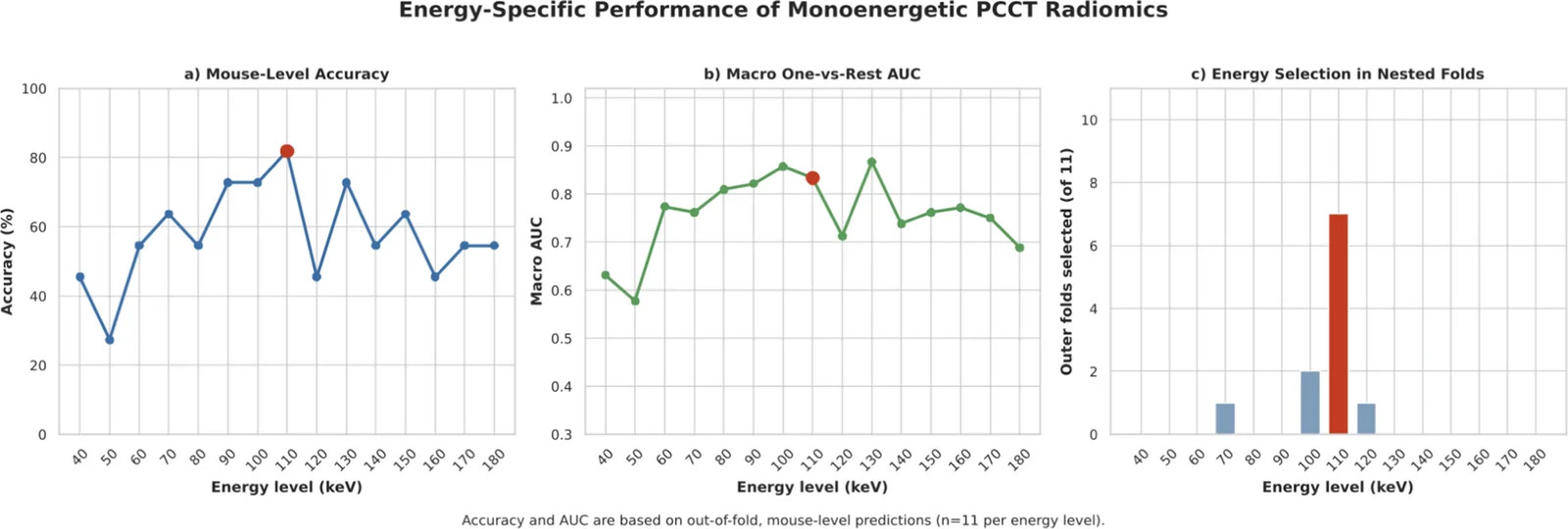
Energy-specific mouse-level classification performance of monoenergetic photon-counting computed tomography (PCCT) radiomics. **a** Mouse-level accuracy and **b** macro one-vs-rest area under the ROC curve (AUC) for monoenergetic reconstructions from 40 to 180 keV. Metrics were calculated from out-of-fold predictions generated by nested leave-one-mouse-out cross-validation, with organ-level class probabilities averaged to obtain one prediction per mouse. **c** Frequency with which each monoenergetic reconstruction energy was selected within the outer-training folds of the fully nested spectral workflow. The 110-keV reconstruction showed the highest mouse-level accuracy and was selected most frequently across outer folds, whereas the highest macro one-vs-rest AUC was observed at 130 keV

Classification performance varied considerably across the 15 monoenergetic reconstruction levels. Mouse-level accuracy ranged from 27.3% to 81.8%. The highest energy-specific accuracy was observed at 110 keV, with 9 of 11 mice correctly classified (81.8%), whereas the highest macro one-vs-rest AUC was observed at 130 keV (AUC = 0.867). Thus, the reconstruction associated with the highest classification accuracy was not identical to that associated with the highest probability-ranking performance. The energy-specific results should therefore be interpreted as an exploratory spectral performance profile rather than as independent confirmatory comparisons. The complete energy-specific performance results are provided in Table 2. Energy-specific accuracy, macro one-vs-rest AUC, and training-only energy-selection frequencies are illustrated in Fig. 5.

**Table 2:** Energy-specific mouse-level age-group classification performance of monoenergetic PCCT reconstructions

Energy (keV)	Correct mice/11	Mouse-level accuracy	Macro OvR AUC
40	5/11	45.5%	0.631
50	3/11	27.3%	0.577
60	6/11	54.5%	0.774
70	7/11	63.6%	0.762
80	6/11	54.5%	0.810
90	8/11	72.7%	0.821
100	8/11	72.7%	0.857
110	9/11	81.8%	0.833
120	5/11	45.5%	0.712
130	8/11	72.7%	0.867
140	6/11	54.5%	0.738
150	7/11	63.6%	0.762
160	5/11	45.5%	0.772
170	6/11	54.5%	0.750
180	6/11	54.5%	0.688

Note: Accuracy and macro one-vs-rest AUC were calculated from out-of-fold mouse-level predictions (n=11 mice per energy level)

Each reconstruction energy was evaluated separately using nested leave-one-mouse-out cross-validation. Accuracy and macro one-vs-rest AUC were calculated from the 11 out-of-fold mouse-level predictions at each energy level. Because all energy levels represent repeated reconstructions of the same 11 mice, these results constitute an exploratory spectral performance profile rather than independent confirmatory comparisons

**Fig. 6:**
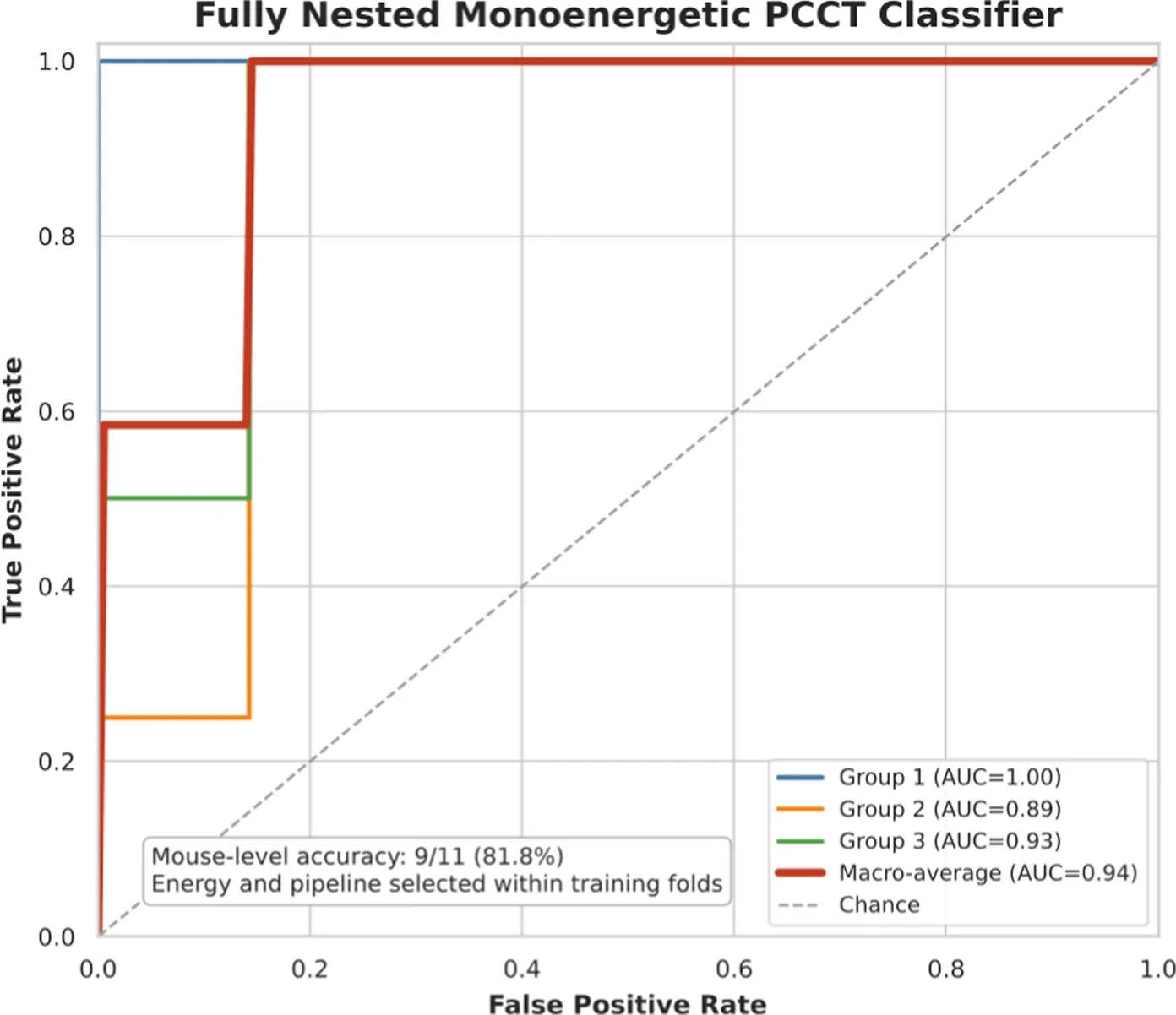
Mouse-level ROC curves for the fully nested monoenergetic PCCT classification workflow. In each outer leave-one-mouse-out fold, all measurements from one mouse were withheld. Reconstruction energy level and classifier pipeline were selected exclusively within the remaining training mice using inner grouped cross-validation. The selected workflow was then refitted on all outer-training mice and applied to the held-out mouse. Organ-level predicted probabilities from heart, liver, and spleen were averaged to derive one mouse-level class probability. The fully nested workflow correctly classified 9 of 11 mice (81.8% mouse-level accuracy)

In the primary fully nested spectral analysis, both reconstruction energy and analytical pipeline were selected exclusively within the outer-training data. This workflow correctly classified 9 of 11 mice, corresponding to a mouse-level accuracy of 81.8%, a balanced accuracy of 83.3%, and a macro-F1 score of 83.3%. The two misclassifications comprised one Group 2 mouse classified as Group 3 and one Group 3 mouse classified as Group 2. The corresponding mouse-level ROC curves for the fully nested monoenergetic workflow are shown in Fig. 6.

The 110-keV reconstruction was selected in 7 of the 11 outer folds and was therefore the most frequently selected energy level. The remaining folds selected 100 keV twice and 70 and 120 keV once each. Pipeline selection also varied across folds: Logistic Regression with PCA was selected in six folds, Random Forest with SelectKBest in four folds, and Logistic Regression with SelectKBest in one fold. These findings indicate an energy-dependent discriminative signal, while also demonstrating that no single reconstruction or analytical pipeline was selected universally across training subsets.

### Mouse-level permutation analysis

To assess whether the observed performance of the fully nested monoenergetic workflow could arise under random age-group assignment, a mouse-level permutation analysis with 200 permutations was performed. The observed accuracy was 81.8% (9/11 mice), whereas the mean accuracy under the empirical null distribution was 27.8% (median, 27.3%; SD, 17.6%). The 95th percentile of the null distribution was 54.5%, and the highest accuracy observed among the 200 permutations was 72.7% (8/11 mice). None of the permuted datasets reached or exceeded the observed accuracy, yielding a plus-one corrected Monte-Carlo p value of 0.005. A detailed summary of the permutation null distribution is provided in Supplementary Table S2.

### Feature-selection stability at 110 keV

**Fig. 7:**
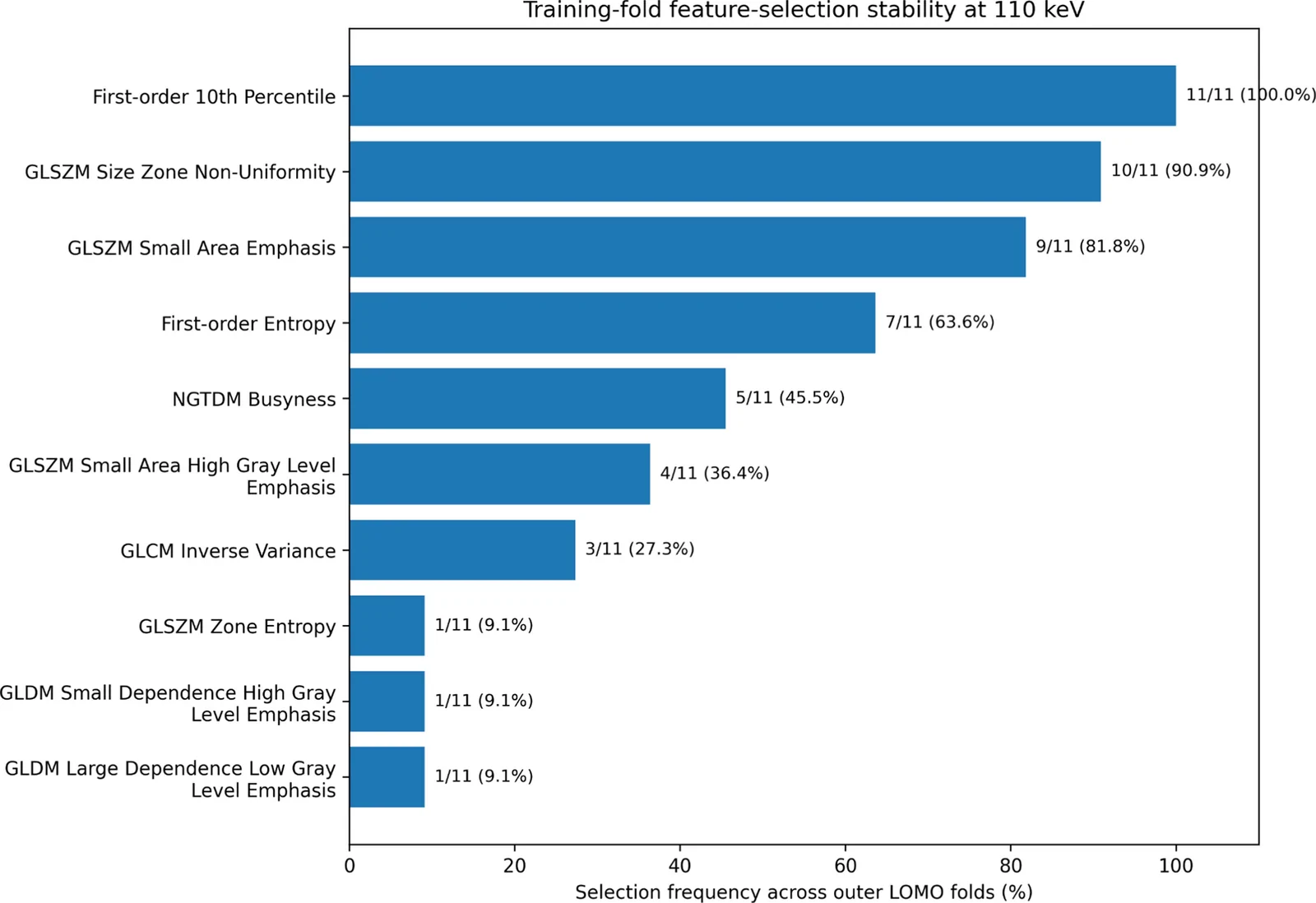
Training-fold feature-selection stability at 110 keV. Feature-selection frequency across the 11 outer leave-one-mouse-out cross-validation folds. Within each fold, correlation filtering and SelectKBest (k=5) were fitted exclusively using the remaining training mice. Bars indicate the proportion of outer folds in which each radiomic feature was selected. This analysis reflects the stability of feature selection across training sets and should not be interpreted as global feature importance or causal biological relevance. The ten most frequently selected radiomic features are shown

Fold-wise feature-selection stability was additionally assessed for the 110-keV reconstruction. First-order 10th Percentile was selected in all 11 outer training folds, followed by GLSZM Size Zone Non-Uniformity in 10/11 folds (90.9%) and GLSZM Small Area Emphasis in 9/11 folds (81.8%). First-order Entropy was selected in 7/11 folds (63.6%), NGTDM Busyness in 5/11 folds (45.5%), GLSZM Small Area High Gray Level Emphasis in 4/11 folds (36.4%), and GLCM Inverse Variance in 3/11 folds (27.3%). These frequencies represent stability of training-fold feature selection and should not be interpreted as global or causal feature importance. The results are displayed in Fig. 7.

## Discussion

The present exploratory study investigated whether CT-derived radiomic signatures contain information associated with chronological age groups in mice and how this information depends on imaging platform and reconstruction strategy. Using the individual mouse as the independent biological unit and nested leave-one-mouse-out cross-validation, classification performance differed substantially between datasets. PCCT-SPP showed the numerically highest performance, correctly classifying 10 of 11 mice (90.9%), followed by the fully nested monoenergetic PCCT workflow with 9 of 11 correctly classified mice (81.8%). In contrast, Albira II achieved substantially lower performance (5/11; 45.5%). These findings indicate that age-associated radiomic discrimination varied substantially across imaging and reconstruction contexts.

Age-related structural, metabolic, and vascular changes have previously been described across multiple murine organs, including heart, liver, and spleen, providing a plausible biological basis for age-associated differences in quantitative imaging features (Chen et al. 2021; Guo et al. 2022; Keele et al. 2023; Scifo et al. 2025). However, the present study did not include an independent biological-aging reference. Accordingly, the observed differences should be interpreted as chronological age-group-associated radiomic phenotypes rather than prediction of biological age.

Monoenergetic PCCT demonstrated marked energy-dependent variation in classification performance. Among individually evaluated energies, the highest mouse-level accuracy was observed at 110 keV (81.8%), whereas the highest macro one-vs-rest AUC occurred at 130 keV (0.867). In the fully nested analysis, 110 keV was selected in 7 of 11 outer folds, while 70, 100, and 120 keV were selected in the remaining folds. Thus, 110 keV was the most frequently selected reconstruction in this cohort, but the current data do not establish a universally optimal energy level. In clinical photon-counting CT, the optimal virtual monoenergetic level has likewise been shown to depend on the specific imaging task (Dunning et al. 2023). Similarly, the numerical difference between PCCT-SPP and the fully nested monoenergetic workflow corresponds to only one mouse and should not be interpreted as evidence of superiority of either reconstruction strategy.

Importantly, the grouped permutation analysis supported that the observed performance of the fully nested monoenergetic workflow was unlikely to arise solely from repeated model optimization under random group assignment. The observed accuracy of 81.8% exceeded all 200 permuted analyses, with a maximum null accuracy of 72.7% and a plus-one corrected Monte-Carlo p value of 0.005. Nevertheless, permutation testing does not establish generalizability and cannot replace validation in an independent cohort.

The revised feature analysis further showed that repeatedly selected features comprised both first-order and texture-based measures. First-order 10th Percentile was selected in all outer training folds, while GLSZM Size Zone Non-Uniformity and GLSZM Small Area Emphasis were selected in 10/11 and 9/11 folds, respectively. These findings indicate consistency of feature selection across training subsets but should not be interpreted as global feature importance, biological causality, or direct evidence of radiomic reproducibility. Although previous studies have reported favorable repeatability of selected PCCT-derived radiomic features (Tharmaseelan et al. 2022; Hertel et al. 2023; Wolf et al. 2023; Zhang et al. 2025), the present study did not include dedicated test–retest imaging or repeated segmentations and therefore cannot independently establish feature stability.

Several limitations require particular consideration. Most importantly, the study included only 11 independent mice across three chronological age groups. Consequently, all performance estimates remain uncertain, and a single misclassified animal changes overall accuracy by approximately 9 percentage points. Heart, liver, and spleen measurements as well as the multiple monoenergetic reconstructions represent repeated measurements within the same animals and do not increase the number of independent biological units. Although organ-level predictions were combined using a prespecified late-fusion approach to generate a single mouse-level prediction, substantial organ-specific radiomic structure remains in the underlying inputs and may interact with age-associated patterns. Dedicated organ-specific analyses in larger cohorts will therefore be required to determine the relative contribution of individual organs to age-group discrimination.

The high dimensionality of the radiomic data relative to the small number of independent animals also creates an inherent risk of model instability and overfitting. This was addressed by substantially reducing model complexity, applying all preprocessing and feature selection exclusively within the respective training folds, and using nested leave-one-mouse-out cross-validation. In addition, reconstruction-energy selection for the primary monoenergetic analysis was performed exclusively within the outer-training data, and grouped mouse-level permutation testing was used to assess whether the observed performance could arise under random class assignment. Nevertheless, neither nested validation nor permutation testing can substitute for validation in an independent external cohort.

Further limitations relate to biological and technical interpretation. No independent reference standard of biological aging was available, limiting interpretation of the identified radiomic patterns beyond chronological age-group associations. The comparison between Albira II and PCCT should be interpreted as a comparison of imaging platforms and protocols, since the systems differ in detector architecture, acquisition geometry, reconstruction characteristics, noise properties, and other technical parameters despite common spatial resampling and intensity discretization (Park et al. 2020; Ayx et al. 2022). Accordingly, differences in classification performance cannot be attributed specifically to photon-counting detector technology.

Finally, the monoenergetic energy-specific analysis involved repeated reconstructions of the same 11 mice and should therefore be regarded as an exploratory spectral performance profile. Although 110 keV was selected most frequently in the fully nested analysis, the selected reconstruction varied across folds and no universally optimal energy can be inferred. Likewise, no formal statistical comparison between PCCT-SPP and the fully nested monoenergetic workflow was performed. Macro-AUC estimates are based on only 11 out-of-fold mouse-level predictions and should therefore be interpreted descriptively. Although all segmentations underwent independent review by a second radiologist, repeated segmentations specifically designed to quantify intra- or inter-reader reproducibility were not performed. These limitations reinforce the exploratory nature of the present findings and the need for larger, independently validated studies incorporating biological-aging reference measures and dedicated reproducibility analyses.

In conclusion, CT-derived radiomic features showed age-group-associated patterns in this exploratory murine cohort, with substantial dependence on imaging platform and reconstruction strategy. PCCT-SPP showed the numerically highest classification performance, while monoenergetic PCCT demonstrated pronounced energy dependence, with 110 keV being the most frequently selected reconstruction rather than a universally optimal energy. These findings are hypothesis-generating and require confirmation in larger independent cohorts incorporating biological-aging reference measures and dedicated reproducibility assessments.

## Supplementary Information

Below is the link to the electronic supplementary material.

Supplementary file1 (DOCX 20 KB)

## Data Availability

Data is available from the corresponding author upon reasonable request.

## Abbreviations

AUC: Area under the curve
LOMO-CV: Leave-one-mouse-out cross-validation
PCCT: Photon-counting computed tomography
PCA: Principal component analysis
RF: Random forest
ROC: Receiver operating characteristic
SPP: Spectral post processing
SVM: Support vector machine
XGBoost: Extreme gradient boosting

## Declarations

### AI assistance disclaimer

Portions of the text in this manuscript were assisted by the use of an artificial intelligence tool (Perplexity, powered by GPT‑5) to improve language clarity and readability. The authors take full responsibility for the content, accuracy, and scientific integrity of the manuscript.
