# Supplementary material for: Age-associated radiomic phenotyping in mice using multi-energy photon-counting and preclinical CT: an exploratory study
Source: Biogerontology. 2026 Oct 15;27(6):178. doi: 10.1007/s10522-026-10524-y (PMC13672618; doi:10.1007/s10522-026-10524-y)
Supplement: Supplementary file 1 [file 10522_2026_10524_MOESM1_ESM.docx]

## **Supplemenary Material**

**Supplementary Table S1:** Mouse-level leave-one-mouse-out cross-validated classification performance of feature selection and dimensionality reduction strategies for Albira radiomics.

| **Method** | **Correct mice / 11** | **Mouse-level accuracy** | **Median features retained after correlation filtering** |
| --- | --- | --- | --- |
| Correlation-filtered radiomics + XGBoost | 5/11 | 45.5% | 327 |
| PCA (5 components) + XGBoost | 4/11 | 36.4% | 327 |
| PCA (10 components) + XGBoost | 4/11 | 36.4% | 327 |
| PCA (15 components) + XGBoost | 4/11 | 36.4% | 327 |
| PCA (20 components) + XGBoost | 3/11 | 27.3% | 327 |
| SelectKBest (10 features) + XGBoost | 3/11 | 27.3% | 327 |
| SelectKBest (20 features) + XGBoost | 3/11 | 27.3% | 327 |
| SelectKBest (30 features) + XGBoost | 4/11 | 36.4% | 327 |
| SelectKBest (50 features) + XGBoost | 3/11 | 27.3% | 327 |

*Note: All preprocessing, PCA, and feature selection were fitted independently within each leave-one-mouse-out (LOMO) training fold. Accuracy was calculated from 11 biologically independent mouse-level predictions; organ-level class probabilities from heart, liver, and spleen were averaged to obtain one prediction per mouse. All configurations represent prespecified secondary sensitivity analyses using the same fixed XGBoost classifier. No XGBoost hyperparameter optimization was performed, and no adaptive selection among these configurations was used to derive the primary study results. The reported median of 327 features refers to the number of features retained after training-fold-specific correlation filtering.*

**Supplementary Table S2:** Mouse-level permutation analysis of the fully nested monoenergetic PCCT classification workflow.

Age-group labels were permuted at the mouse level while preserving the original group-size distribution of 3/4/4 mice. For each of 200 permutations, the complete nested analytical workflow was repeated, including training-fold preprocessing, feature selection, inner pipeline selection, and reconstruction-energy selection. The observed mouse-level accuracy of 81.8% (9/11 mice) exceeded the maximum accuracy obtained under permutation (72.7%; 8/11 mice). None of the 200 permuted datasets reached or exceeded the observed performance. The Monte-Carlo p-value was calculated using the plus-one correction, (b + 1)/(B + 1), yielding p = 0.005.

| **Permutation-analysis metric** | **Result** |
| --- | --- |
| Observed accuracy | 81.8% (9/11) |
| Number of permutations | 200 |
| Null mean | 27.8% |
| Null median | 27.3% |
| Null SD | 17.6% |
| Null 95th percentile | 54.5% (6/11) |
| Maximum permuted accuracy | 72.7% (8/11) |
| Permutations ≥ observed | 0/200 |
| Monte-Carlo p-value | 0.005 |

| **Correcly classified mice** | **Number of permutations** |
| --- | --- |
| 0/11 | 19 |
| 1/11 | 28 |
| 2/11 | 39 |
| 3/11 | 34 |
| 4/11 | 30 |
| 5/11 | 29 |
| 6/11 | 13 |
| 7/11 | 4 |
| 8/11 | 4 |
| 9/11 | 0 |

**Supplementary Table S3.** Candidate model configurations and hyperparameter settings for the Albira II and PCCT-SPP analyses.

| **Candidate pipeline** | **Parameters evaluated** | **Fixed settings** |
| --- | --- | --- |
| Logistic Regression + SelectKBest | k = 3, 5; C = 0.1, 1.0 | class_weight = balanced; max_iter = 5,000; random_state = 42 |
| RBF-SVM + SelectKBest | k = 3, 5; C = 0.1, 1.0 | gamma = scale; class_weight = balanced; probability = True; random_state = 42 |
| Random Forest + SelectKBest | k = 3, 5; max_depth = 3, 8; min_samples_leaf = 1, 2 | n_estimators = 50; min_samples_split = 2; class_weight = balanced_subsample; random_state = 42 |
| Logistic Regression + PCA | explained variance = 80%, 90%; C = 0.1, 1.0 | class_weight = balanced; max_iter = 5,000; random_state = 42 |
